# Correlation between MMP2 expression in lung cancer tissues and clinical parameters: a retrospective clinical analysis

**DOI:** 10.1186/s12890-020-01317-1

**Published:** 2020-10-28

**Authors:** Liping Han, Baowei Sheng, Qingdi Zeng, Wei Yao, Qiufang Jiang

**Affiliations:** 1 Department of Respiration, Jining NO.1 People’s Hospital, Jining, China; 2 Affiliated Jining NO.1 People’s Hospital of Jining Medical University, Jining Medical University, Jining, China; 3 Department of Clinical Laboratory, Jining NO.1 People’s Hospital, Jining, China; 4 General Surgery, Zoucheng Kanzhuang Township Health Center, Zoucheng, China

**Keywords:** Lung cancer, Matrix metalloproteinase 2, MMP2, Diagnosis, Prognosis

## Abstract

**Background:**

Matrix metalloproteinase 2 (MMP2) has been found to be related to malignant tumors; the aim of this study was to investigate the correlation between MMP2 expression in lung cancer tissues and clinical parameters of lung cancer.

**Methods:**

The expression of MMP2 in lung cancer tissues and in adjacent non-malignant tissues was tested by immunohistochemistry. The correlation between the expression of MMP2 and clinical parameters of lung cancer was analyzed by Kaplan-Meier curve and multiple regression analysis.

**Results:**

The expression of MMP2 was higher in lung cancer tissues than that in adjacent non-malignant tissues (*p* = 0.002). Increased MMP2 was associated with low differentiation (*p* = 0.022), tumor size (*p* = 0.032), lymph node metastasis (*p* < 0.001), advanced stage (*p* = 0.002). The post-surgical survival time in patients with high MMP2 expression was shorter than that in patients with low MMP2 expression (*p* = 0.001). High expression of MMP2 (*p* = 0.006) and advanced stage (*p* = 0.003) were independent prognostic indicators for survival of lung cancer patients.

**Conclusions:**

Increased MMP2 correlates with malignant biological behavior of lung cancer and it could be a potential biomarker for diagnosis and prognosis of the disease.

## Introduction

Lung cancer is divided into non-small cell lung cancer (NSCLC) and small cell lung cancer (SCLC) in clinical practice [1]. Most lung cancers are NSCLC, which is significantly different from SCLC in pathology, pathogenesis and prognosis [2]. It has been confirmed that many molecular signals are involved in the occurrence and development of lung cancer, which are often related to the abnormal expressions of some proteins. The research on proteomics and genomics has brought a new perspective for the prevention and treatment of lung cancer [3]. Extracellular matrix (ECM) is a complex network structure composed of macromolecular substances secreted by cells into the extracellular matrix. ECM supports and connects tissue structure, and it participates in regulating tissue generation, cell metabolism and signal transduction [4]. Matrix metalloproteinases (MMPs) are enzymes that rely on metal ions such as zinc and calcium to perform their biological functions. It can degrade various protein components in ECM, destroy the histological barrier of tumor cell invasion, and play a key role in tumor invasion and metastasis [5–7].

Matrix metalloproteinase 2 (MMP2) is one of the members of MMPs. The gene sequence encoding MMP2 contains 13 exons and 12 introns [8]. The protein size of MMP2 gene translation is about 72 kDa. MMP2 is a zinc-dependent enzyme that can cut extracellular matrix components [9]. It is suggested that MMP-2 is related to the degradation of ECM and plays a role in tumor cell growth, differentiation, invasion, metastasis, regulation of tumor angiogenesis and immune surveillance [10]. Up-regulation of MMP2 has been found in many tumors, and its increase promotes the proliferation, motility and metastasis of malignant tumor cells [11–15]. However, down-regulating the expression of MMP2 can reduce tumor cell proliferation, clonal growth and metastasis, and promote tumor cell apoptosis [16–18]. This study detected the expression of MMP2 in lung cancer tissues and analyzed the correlation between its expression level and the clinical characteristics of lung cancer.

## Material and methods

### Patients

From January 2010 to December 2012, 65 patients were diagnosed with lung cancer and underwent lung cancer resection at the Jining NO.1 People’s Hospital, Jining, China. The lung cancer tissues and the matching adjacent non-malignant tissues (from the edge of the tumor block at least 3 cm) from these patients were collected for a retrospective study. The clinicopathological data of patients are listed in Table 1. All patients were approached based on approved ethical guidelines, and those who agreed to participate in this study were required to sign consent forms. The study was approved by Research Ethics Committee of Jining NO.1 People’s Hospital, Jining, China.

**Table 1:** Clinico-pathological features of patients involve in the study

	Lung cancer patients for tissues test	
Items	Characteristics	Lung cancer patients (*N* = 65)
Gender	Male	51 (78.5%)
	Female	14 (21.5%)
Ages	< 60	33 (50.8%)
	≥60	32 (49.2%)
Smoking	Yes	34 (52.3%)
	No	31 (47.7%)
Histology	LAC	25 (38.5%)
	LSCC	31 (47.7%)
	SCLC	9 (13.8%)
Differentiation degree	Poorly	19 (29.2%)
	Moderately	23 (35.5%)
	Well	14 (21.5%)
	Unavailable	9 (13.8%)
T staging	T1-T2	37 (56.9%)
	T3-T4	19 (29.2%)
	Unavailable	9 (13.9%)
Lymphatic metastasis	N0 - N1	23 (35.4%)
	N2 - N3	33 (50.8%)
	Unavailable	9 (13.8%)
Clinical staging	I-II	36 (55.4%)
	III-IV	20 (30.8%)
	Unavailable	9 (13.8%)

*LAC* lung adenocarcinoma, *LSCC* lung squamous cell carcinoma, *SCLC* small cell lung cancer, *N* the grade of lymphatic invasion

### Tissue microarray (TMA) construction

A tissue array instrument was employed to construct the tissue microarray (TMA) (Beecher Instruments, Manual Tissue Arrayer, USA). The representative tumor area on donor wax blocks was screened and the tissue core of 2 mm was perforated from the labeled donor tissue. The tissue core collected was placed into the small hole of the recipient wax mass. Repeatedly, the tissue cores were all placed according to the order of design. After dehydrating and fixing, the TMA block was cut into slices as a thickness of 4.5 μm. Then, the tissue slices were attached to poly-lysine-treated slides and were stored at − 4 °C.

### Immunohistochemistry (IHC)

The expression of MMP2 was determined using SABC IHC techniques (SA1094, Bostere Biotech Company, Wuhan, China) according to the introduction of kit. A rabbit anti-human MMP2 monoclonal antibody (BA3716; 1:50 dilution; Bostere Biotech Company, Wuhan, China) was used as the first antibody. The slices of positive staining provided by the antibody kit were served as the positive control. The phosphate buffer saline (PBS) in stead of the first antibody was used as the negative control. Staining intensity and staining area were two semi-quantitative indicators of MMP2 staining. Immunostaining was blindly assessed according to previously published scoring methods [19]. The staining intensity includes the following levels: 0, no staining; 1, mild staining; 2, moderate staining; 3, intense staining. The score for the staining area is as follows: 0, no staining in any fields; 1, < 30% of staining area, 2, between 30 and 60% staining area; 3, > 60% staining area. A combined staining score (intensity + area) of ≤2 was defined as low expression, between 3 and 4 as moderate, and between 5 and 6 was considered as high expression [19].

### Statistical analysis

The relationship between the expression of MMP2 in tissues and clinicopathological parameters was analyzed by the Chi-square test, McNemar test and Fisher’s exact test. The definition of patient’s survival is from the date of the operation to the date of death (the death of any cause) or the last known date of the patient’s life (loss of visit). The survival curve was drawn according to kaplan-meier method. The logarithmic rank test was used to determine whether the expression of MMP2 affect the survival of patients. A Cox-proportional risk regression model with multivariate analysis was employed to test the mixed effect of variables with the most closely correlated with the expression levels of MMP2. All tests were two-sided. A statistical significance was that the *P* value was less than 0.05. The statistical analysis was performed by the SPSS 19.0 software [20].

## Results

### Expression of MMP2 in lung cancer tissues was higher than that in adjacent normal tissues

**Fig. 1:**
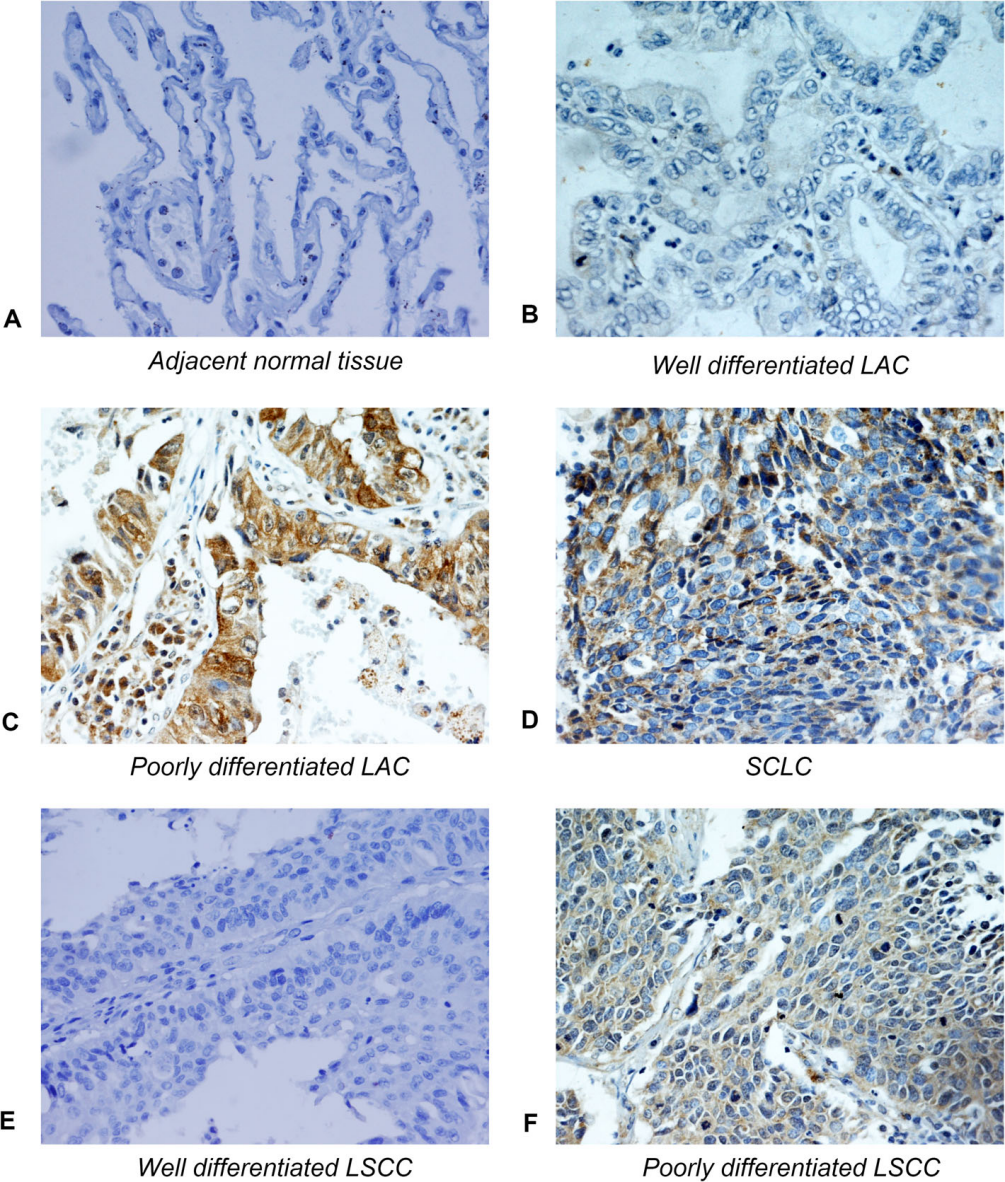
IHC analysis of MMP2 in lung cancer and normal tissues (IHC × 400). **a** Low expression of MMP2 in normal tissues. **b** Low expression of MMP2 in well differentiated LAC. **c** High expression of MMP2 in poorly differentiated LAC. **d** High expression of MMP2 in SCLC. **e** Low expression of MMP2 in well differentiated LSCC. **f** High expression of MMP2 in poorly differentiated LSCC. MMP2, matrix metalloproteinase 2; SCLC, small cell lung cancer. LAC, lung adenocarcinoma. LSCC, lung squamous cell carcinoma

**Fig. 2:**
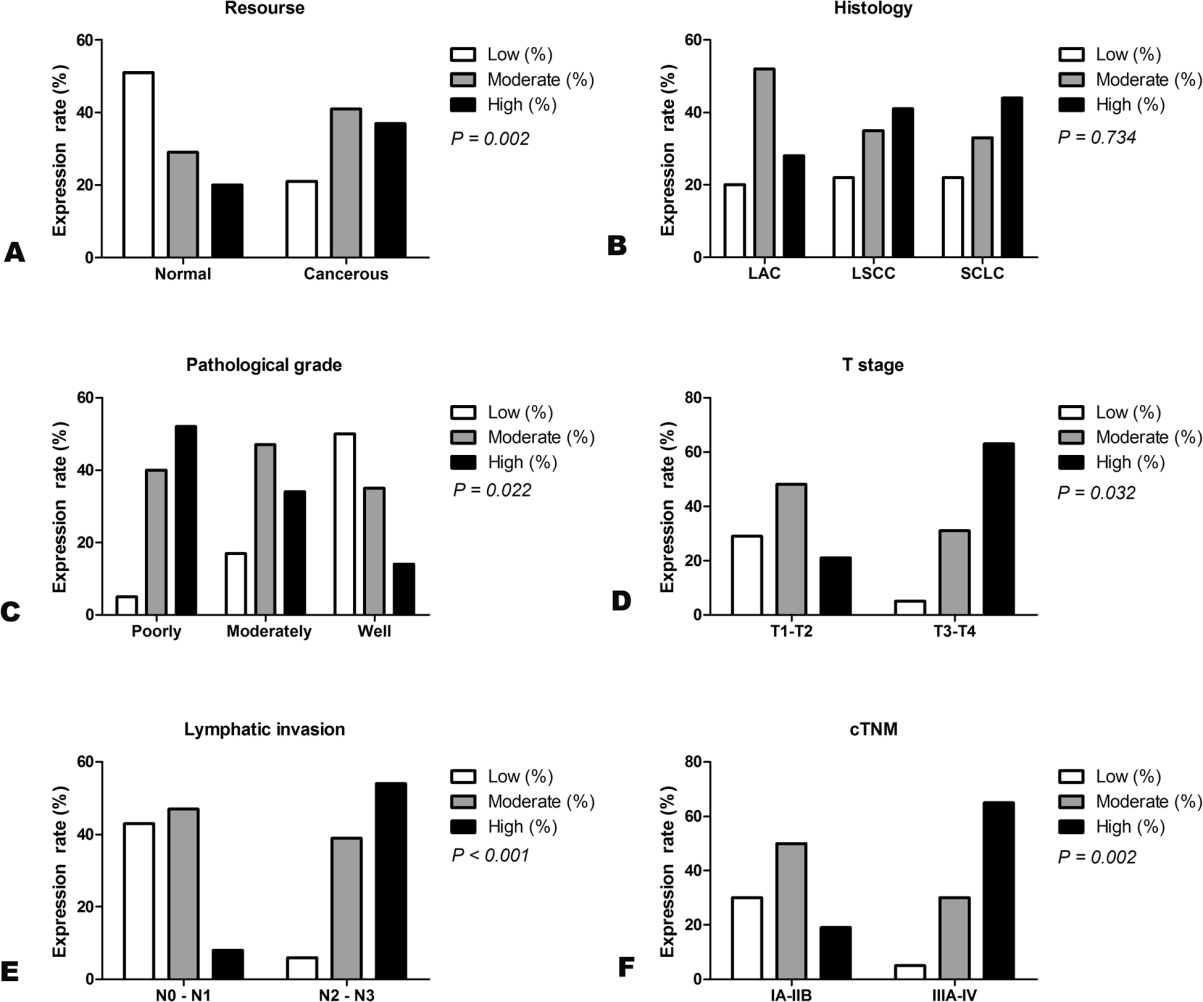
Correlation between the clinico-pathological features and MMP2 expression in lung cancer tissues. **a** MMP2 was highly expressed in lung cancer tissues, whereas it was lowly expressed in matched adjacent non-malignant tissues (*p* < 0.05). **b** Expression of MMP2 did not relate to the histological type of lung cancer patients (*p* > 0.05). **c** Upregulation of MMP2 was observed in poorly differentiated lung cancer tissues compared with well-differentiated tissues (*p* < 0.05). **d** Expression of MMP2 in lung cancer tissues at T3–4 stage was significantly higher than that in T1–2 stage (*p* < 0.05). **e** MMP2 expression in lung cancer tissues with N0-N1 of lymphnode metastasis was lower than that in those with N2-N3 of lymph node metastasis (*p* < 0.05). **f** Increase of MMP2 was displayed in tissues with lung cancer at stages III-IV compared with those at stages I-II (*p* < 0.05). MMP2, matrix metalloproteinase 2; SCLC, small cell lung cancer. LAC, lung adenocarcinoma. LSCC, lung squamous cell carcinoma. N, node stage (TNM classification)

In IHC analysis, MMP2 was mainly expressed in cytoplasm and membrane of lung cancer cells and normal cells (Fig. 1a-f). Statistical analysis showed that high expression rate of MMP2 reached 37% (24/65) in lung cancer tissues, whereas only accounted for 20% (13/65) in the adjacent non-malignant tissues, which suggested that the expression of MMP2 in lung cancer tissues was higher than that in adjacent normal tissues (*p* = 0.002) (Table 2, Fig. 2a).

**Table 2:** Expression of MMP2 in lung cancer tissues (*N* = 65)

Parameter	Group	N	Expression of MMP2				
			Low (%)	Moderate (%)	High (%)	χ^2^ value	*P* value
Category	Normal	65	33 (50.8)	19 (29.2)	13 (20)	12.342	0.002
	Cancerous	65	14 (21.5)	27 (41.5)	24 (37)^●^		
Gender	Male	51	13 (25.5)	20 (39.2)	18 (35.3)	2.195	0.334
	Female	14	1 (7.1)	7 (50)	6 (42.9)		
Ages	< 60	33	7 (21.2)	15 (45.5)	11 (33.3)	0.482	0.785
	≥60	32	7 (21.9)	12 (37.5)	13 (40.6)		
Smoking	Yes	34	9 (26.5)	13 (38.2)	12 (35.3)	1.044	0.593
	No	31	5 (16.1)	14 (45.2)	12 (38.7)		
Histology	LAC	25	5 (20)	13 (52)	7 (28)	2.011	0.734
	LSCC	31	7 (22.6)	11 (35.5)	13 (41.9)		
	SCLC	9	2 (22.2)	3 (33.3)	4 (44.4)		
Pathological grade	Poorly	19	1 (5.3)	8 (42.1)	10 (52.6)^★^	11.46	0.022
	Moderately	23	4 (17.4)	11 (47.8)	8 (34.8)		
	Well	14	7 (50)	5 (35.7)	2 (14.3)		
	Unavailable	9	2 (22.2)	3 (33.3)	4 (44.4)		
T staging	T1-T2	37	11 (29.7)	18 (48.6)	8 (21.6)	10.567	0.032
	T3-T4	19	1 (5.3)	6 (31.6)	12 (63.2)^▲^		
	Unavailable	9	2 (22.2)	3 (33.3)	4 (44.4)		
Lymph node metastasis	N0 - N1	23	10 (43.5)	11 (47.8)	2 (8.7)	17.06	< 0.001
	N2 - N3	33	2 (6.1)	13 (39.4)	18 (54.5)^■^		
	Unavailable	9	2 (22.2)	3 (33.3)	4 (44.4)		
pTNM	IA-IIB	36	11 (30.6)	18 (50)	7 (19.4)	12.59	0.002
	IIIA-IV	20	1 (5)	6 (30)	13 (65)^◆^		
	Unavailable	9	2 (22.2)	3 (33.3)	4 (44.4)		

N, numbers of patients; ^●^*p* < 0.05, cancerous tissues compared with normal tissues; ^★^*p* < 0.05, Poorly differentiated tissues compared with well differentiated tissues; ^▲^*p* < 0.05, T3–4 compared with T1–2; ^■^*p* < 0.05, N2-N3 of lymph node metastasis compared with N0-N1; ^◆^*p* < 0.05, IIIA-IV compared with IA-IIB; LAC, lung adenocarcinoma; LSCC, lung squamous cell carcinoma; SCLC, small cell lung cancer

### Highly expressed MMP2 was associated with differentiation, tumor size, lymph node metastasis and clinical stage of lung cancer

Expression of MMP2 was not related to the sex, age, smoking status and histology of lung cancer (*p* > 0.05) (Table 2, Fig. 2b). However, poorly differentiated lung cancer showed a highly expressed MMP2 (10/19, 52.6%) compared with well differentiated lung cancer (2/14, 14.3%) (*p* = 0.022) (Table 2, Fig. 2c). Expression of MMP2 in lung cancer at T3–4 stage (12/19, 63.2%) was higher than that in lung cancer at T1–2 stage (8/37, 21.6%) (*p* = 0.032) (Table 2, Fig. 2d). Expression of MMP2 in lung cancer with N2-N3 (18/33, 54.5%) of lymph node metastasis was up-regulated compared with that in lung cancer with N0-N1 (2/23, 8.7%) (*p* < 0.001) (Table 2, Fig. 2e). Increased MMP2 was displayed in lung cancer tissues at stages IIIA-IV (13/20, 65%) compared with those of stages IA-IIB (7/36, 19.4%) (*p* = 0.002) (Table 2, Fig. 2f).

### Increased MMP2 in lung cancer tissues was related to shorter survival of lung cancer patients

**Fig. 3:**
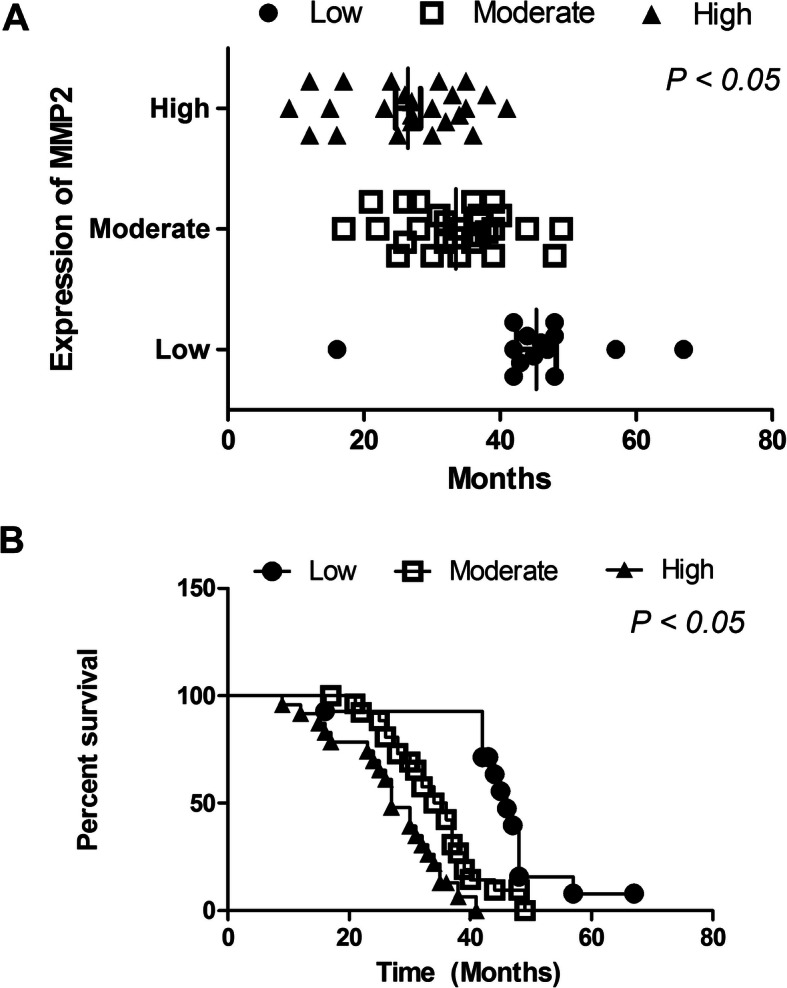
Relationship between MMP2 expression in lung cancer tissues and the survival time of lung cancer patients. **a** The average survival time for patients with low expression of MMP2 was 45.35 ± 10.85 months and for patients with high expression 26.46 ± 8.92 months. **b** The Kaplan-Meier curves suggested that post-surgical survival time in patients with high MMP2 expression was shorter than that in patients with low MMP2 expression. MMP2, matrix metalloproteinase 2

According to the different expression intensity of MMP2 in patients, it is divided into three cohorts: patients with low expression, patients with moderate expression and patients with high expression. As shown in Table 3, the average survival time for patients with low expression of MMP2 was 45.35 ± 10.85 months (95% Cl: 39.1 to 51.6), for patients with moderate expression was 33.52 ± 7.83 months (95% CI: 30.4 to 36.6) and for patients with high expression was 26.46 ± 8.92 months (95% CI: 22.7 to 30.2). The Kaplan-Meier curves suggested that post-surgical survival time in patients with high MMP2 expression was shorter than that in patients with low MMP2 expression (*p* = 0.001) (Fig. 3e and f).

**Table 3:** Correlation between MMP2 expression and survival time of lung cancer patients

	Expression	N	Mean (months)	Std. Deviation (months)	Std. Error	95% Confidence Interval	
						Lower Bound	Upper Bound
MMP2	Low	14	45.3571	10.85291	2.90056	39.0909	51.6234
	Moderate	27	33.5185	7.82683	1.50627	30.4223	36.6147
	High	24	26.4583	8.92471	1.82175	22.6898	30.2269

*CI* confidence interval, *MMP2* matrix metalloproteinase 2

### High expression of MMP2 and advanced stage were independent prognostic indicators for survival of lung cancer patients

A total of 9 variables, gender (X1), age (X2), smoking (X3), histology (X4), differentiation (X5), lymphatic invasion (X6), T stage (X7), TNM (X8), MMP2 (X9)), were included in the multiple regression analysis. The results of analysis showed that high expression of MMP2 (*p* = 0.006) and advanced stage (*p* = 0.003) were introduced into regression equation, which means they could be independent prognostic indicators for survival of patients (*p* < 0.05). The final risk function was H (t) = [h_0_ (t)] e ^(2.01X8 + 1.49X9)^ (Table 4).

**Table 4:** Multiple regression analysis of the relationship between MMP2 and lung cancer

Variables (X)	Categories (different groups)	*P* value	OR value	95% CI for OR	
				lower	upper
Gender (X1)	Male (X_1–0_) vs. female (X_1–1_)	0.154	0.532	0.223	1.267
Age (X2)	< 60 (X_2–0_) vs. ≥60 (X_2–1_)	0.618	0.808	0.422	1.671
Smoking (X3)	No (X_3–0_) vs. Yes (X_3_–_2_)	0.558	0.810	1.401	1.637
Histology (X4)	LAC (X_4–0_) vs. LSCC (X_4_–_1_) vs. SCLC (X_4_–_2_)	0.300	0.702	0.360	1.370
Differentiation (X5)	Poor (X_5–0_) vs. moderate (X_5–1_) vs. well (X_5–2_)	0.633	0.620	0.704	1.781
Lymphatic invasion (X6)	N0-N1(X_6–0_) vs. N2-N3 (X_6–1_)	0.540	0.485	0.048	4.919
T stage (X7)	T1-T2 (X_7–0_) vs. T3-T4 (X_7–1_)	0.307	0.648	0.139	12.32
TNM (X8)	IIA - IIIA (X_8–0_) vs. IIIB - IV (X_8–1_)	0.003	2.01	2.013	3.078
MMP2 (X9)	Low (X_9–0_) vs. moderate (X_9–1_) vs. high (X_9–2_)	0.006	1.49	1.012	2.876
Risk function:		H(t) = [h_0_(t)] e ^(2.01 X8 + 1.49X9)^			

*LAC* lung adenocarcinoma, *LSCC* lung squamous cell carcinoma, *SCLC* small cell lung cancer, *TNM* clinical stage of lung cancer, *OR* odds ratio, *MMP2* matrix metalloproteinase 2, *CI* confidence interval

## Discussion

Compared with normal cells and tissues, the expression of some proteins is often upregulated in lung cancer cells and tissues. Moreover, these up-regulated proteins often have a certain inherent relationship with the malignant biological behavior of lung cancer. So, identification of the abnormal proteins in lung cancer may be of great value in the developing of lung cancer novel diagnostic and therapeutic methods [3]. There is substantial evidence that MMP2 plays a major role in tumor cell-mediated degradation of extracellular matrix and its expression is closely related to the invasion, metastasis and prognosis of human malignant tumors [8, 10, 11, 15]. By testing the expression level of MMP2 in lung cancer tissues, we analyzed the correlation between the MMP2 and the biological behavior of lung cancer. We found that the MMP2 was highly expressed in lung cancer tissues but not in adjacent normal tissues, suggesting that high expression of MMP2 might potentially related to lung cancer occurrence and development. Our findings are consistent with previous reports that MMP2 is highly expressed in lung cancer tissues and lowly expressed in normal tissues [21, 22].

It is well known that the degree of differentiation of lung cancer cells is an independent factor affecting the prognosis of lung cancer patients, which is mainly based on that poorly differentiated lung cancer cells have a high degree of malignancy, and will soon be transferred and relapsed. In our study, we found that MMP2 was highly expressed in poorly differentiated lung cancer tissues, indicating that MMP2 may be associated with the malignant degree of lung cancer. We also found that increased MMP2 in lung cancer tissues correlated with lymph node metastasis and advanced TNM stage. In addition, we noticed that with the increase of tumor volume, the expression of MMP2 showed an increasing trend. These results showed a clear clue that determining the expression of MMP2 could be a valuable strategy for predicting metastatic potential and prognosis of lung cancer. This inference can also be testified by previous research conclusions that MMP2 is associated with the molecular classification of some malignant tumors and has a close relationship with invasiveness, proliferation, lymphatic metastasis, and poor prognosis [8, 11, 12, 15], and our findings are similar to this. This is a positive view that TNM stage correlates with prognosis of cancer patients, which is mainly based on lymph node metastasis, tumor size, and distant metastasis. Our study showed that increased MMP2 significantly correlated with the malignant features of lung cancer such as tumor size, invasion and metastasis of lymph nodes, low differentiation and advanced stage. The findings suggested that MMP2 could be used as a diagnostic and prognostic indicator of lung cancer. Existing literature shows MMP2 not only degrades the basement membrane and matrix to break through the matrix barrier, thus promoting tumor invasion and metastasis, but also accelerates tumor growth and proliferation through neovascularization [9]. Activated transcription factor 1 (ATF1) plays an important role in the migration and invasion of lung cancer cells. A study proposed that the silencing of ATF1 can induce the down-regulation of MMP2 expression, thereby inhibiting the migration and invasion of lung cancer cells [23]. Recent publications and clinical trials have emphasized the potential value of measuring circulating or tissue MMP-2 levels as a tool for tumor diagnosis or prognosis prediction, or as a primary therapeutic target. However, the application of these conclusions to patients is still uncertain, but it is undoubtedly worthy of further efforts [9].

Through the COX regression analysis, we found that lung cancer patients with high-expression of MMP2 had a shorter survival time compared with those with low-expression. In addition, we also found that advanced TNM stage was to be a strong risk factor for shorter survival time of lung cancer patients, with a hazard ratio value of 2.01. In our study, these two factors were implicated into the regression equation and the regression equation was as H(t) = [h_0_(t)] e ^(2.01X8 + 1.49X9)^, which suggested that determining the expression of MMP2 in lung cancer tissues is helpful for predicting the prognosis of lung cancer patients. Our conclusion is similar to previous studies that the high expression of MMP2 in NSCLC was associated with tumor type and clinical stage and has a predictive value for low survival rates, short overall survival (OS) and disease free survival (DFS) [21, 24, 25]. Increased MMP2 correlates with histo-differentiation, lymphatic metastasis, progression, and prognosis of lung cancer, suggesting that it could reflect the situation of progression and migration of lung cancer to a certain extent and that it could be a useful diagnostic, prognostic, and predictive biomarker of lung cancer. A previous study suggests that the expression of MMP2 in lung squamous cell carcinoma is higher than that in lung adenocarcinoma, and its high expression is closely related to lymph node metastasis and TNM staging, and affects the prognosis of patients [21]. However, another study suggests that MMP2 protein has nothing to do with the histology of NSCLC, but its level correlates with poorly differentiated tissue and distant metastasis [22]. These results indicate that the expression of MMP2 in lung cancer is still controversial.

However, There are opinions that MMP2 suppression decreases the expression of hypoxia inducible factor-HIF-1α (HIF-1α) and disrupts phosphoinosmde-3-kinase (PI3K) dependent vascular endothelial growth factor (VEGF) expression, and A549 xenograft tissue sections from mice that treated with MMP-2 small interfering RNA shows reduced expressions of VEGF, providing new insights into the mechanisms underlying MMP2-mediated VEGF expression in lung tumor angiogenesis [26]. Another new study suggests that silibinin inhibits the proliferation, migration, and invasion of triple-negative breast cancer cells by down-regulating the signal of MMP2 [27]. Based on the above results, we can think that MMP2 is associated with the occurrence and development of NSCLC, but the relationship between the two is still certainly unclear. In the future, more multi-center, large-scale studies are still needed to solve this problem. More importantly, the intrinsic mechanism of MMP2 affecting the development of NSCLC remains unclear, A lot of work still needs to be done in this area in the future. There are still some shortcomings in this study. Firstly, most patients of the surgical resection belong to patients with clinical stage IIIB and before, which may lead to a selective bias of the specimen. Secondly, the number of patients included is relatively small. Thirdly, this study did not concern the specific mechanisms by which MMP2 affects the growth, proliferation and metastasis of lung cancer cells. Hence, further experiments involving the mutual regulation-mechanism of MMP2 in lung cancer cells should be done.

## Conclusions

First, the expression of MMP2 was increased in lung cancer tissues and increased MMP2 correlated with poorly differentiated cancer, tumor size, lymphatic metastasis and advanced TNM stage. In addition, increase of MMP2 in lung cancer tissues was correlated with shorter survival of patients. Moreover, highly expressed MMP2 is an independent risk factor for poor prognosis of lung cancer patients, suggesting that MMP2 could be a useful diagnostic, prognostic, and predictive biomarker of lung cancer.

## Data Availability

The datasets used and/or analysed during the current study are available from the corresponding author on reasonable request.

## Abbreviations

ABC: Avidin-biotin-peroxidasecomplex
ATF1: Activated transcription factor 1
DFS: Disease free survival
ECM: Extracellular matrix
EGFR: Epidermal growth factor receptor
HIF-1α: Hypoxia inducible factor-1α
IHC: Immunohistochemistry
MMP2: Matrix metalloproteinase 2
MMPs: Matrix metalloproteinases
NSCLC: Non-small cell lung cancer
OS: Overall survival
PBS: Phosphate buffer saline
PI3K: Phosphoinosmde-3-kinase
SCLC: Small cell lung cancer
TMA: Tissue microarray
TMB: Tetramethylbenzidine
VEGF: Vascular endothelial growth factor
